# Systematic Review of Focal Prostate Brachytherapy and the Future Implementation of Image-Guided Prostate HDR Brachytherapy Using MR-Ultrasound Fusion

**DOI:** 10.1155/2016/4754031

**Published:** 2016-05-16

**Authors:** M. Sean Peach, Daniel M. Trifiletti, Bruce Libby

**Affiliations:** Department of Radiation Oncology, University of Virginia School of Medicine, Charlottesville, VA 22908, USA

## Abstract

Prostate cancer is the most common malignancy found in North American and European men and the second most common cause of cancer related death. Since the practice of PSA screening has become common the disease is most often found early and can have a long indolent course. Current definitive therapy treats the whole gland but has considerable long-term side effects. Focal therapies may be able to target the cancer while decreasing dose to organs at risk. Our objective was to determine if focal prostate brachytherapy could meet target objectives while permitting a decrease in dose to organs at risk in a way that would allow future salvage treatments. Further, we wanted to determine if focal treatment results in less toxicity. Utilizing the Medline repository, dosimetric papers comparing whole gland to partial gland brachytherapy and clinical papers that reported toxicity of focal brachytherapy were selected. A total of 9 dosimetric and 6 clinical papers met these inclusion criteria. Together, these manuscripts suggest that focal brachytherapy may be employed to decrease dose to organs at risk with decreased toxicity. Of current technology, image-guided HDR brachytherapy using MRI registered to transrectal ultrasound offers the flexibility and efficiency to achieve such focal treatments.

## 1. Introduction

Radiation therapy (RT) for prostate cancer came into common use in the United States utilizing an external beam approach during the 1920s. However, disease was often inadequately treated due to poor tissue penetration along with significant dermal morbidity produced by the treatment [1]. Combined with the development of androgen therapy there was lost interest in RT. Additionally, there was a decrease utilization of brachytherapy for all disease sites in the first half of the 20th century due to the significant radiation exposure to nurses and physicians. These shortcomings were overcome by the development of low activity radioactive iodine seeds that required minimal shielding during handling and, once placed in patients, released a negligible dose to the outside environment [2]. These seeds were first successfully employed for prostate cancer at Memorial Sloan Kettering in 1967, with hundreds of subsequent cases in the 1970s using the Whitmore open hand technique. Briefly, a laparotomy was performed and the prostate was mobilized from all but the posterior rectal facing wall. Evenly spaced empty catheters were then placed through the exposed prostate until felt by the physician rectally, followed by permanent seed placement. It was determined that doses greater than 100 Gy produced a five-year disease-free survival rate of 80%. However, there were considerable long-term issues with inadequate coverage and morbidity from the invasive nature of the procedure; thus the technique was abandoned by the mid 1980s.

At the time that the free hand technique was in decline there were several advancements made in diagnostic imaging that would have profound impact on prostate brachytherapy. Holm was able to combine the application of ultrasound (US) guided seed placement for pancreatic cancer with transrectal US (TRUS) to develop US guided low dose rate (LDR) prostate brachytherapy. In Europe this was combined with external beam radiation therapy (EBRT) on a series of patients. However the high dose (160 Gy LDR and 46 Gy EBRT) resulted in significant side effects [3]. In the early 1990s this technique was refined by utilizing lower doses, achieving outcomes similar to prostatectomy with less overall side effects [4]. By the mid 1990s high dose rate brachytherapy (HDR) was practiced at a handful of centers, which offered an additional degree of conformity by being able to shape the delivered dose based on computer optimization of source dwell times. As a result of these developments prostate brachytherapy either as monotherapy or as part of a combined EBRT regimen has become a mainstream therapeutic option for low risk and intermediate risk prostate cancer [5].

There are significant risks of side effects associated with either surgery or RT, including urinary toxicity, erectile dysfunction, and, in the case of RT, rectal toxicity. In the pre-PSA era, prostate disease was not detected until it was more advanced, requiring treatment of the entire gland and immediate periphery to control disease and impart increased survival. Thus such symptoms were viewed as an acceptable necessity. With the advent of PSA screening in the 1990s and a better understanding of the behavior of prostate cancer, disease in the modern PSA era is found significantly earlier and typically has a more indolent course [6]. In patients with low risk disease active surveillance is an acceptable option, as well as prostatectomy or brachytherapy +/− EBRT [5]. However, for younger patients with isolated lesions a more ideal treatment would be to treat the focus of disease without affecting the urethra or neurovascular bundles which cause the most detrimental side effects of traditional therapies, namely, genitourinary toxicity and erectile dysfunction (ED). These treatment related issues become increasingly significant as the average life span of the population increases, with future patients living increasingly longer with whatever side effects treatment imparts. With advances in imaging modalities partial prostate brachytherapy may be applied with limited toxicity to what is a large patient population who want a temporary measure, with low side effects, but still affords salvage RT or surgery in the future if needed.

In this paper, we perform a systematic review to explore the up-to-date dosimetric and clinical studies of focal and focused prostate brachytherapy. We postulate that focal brachytherapy may offer a salvageable therapy that provides lower side effects. Given the dosimetric advantages of HDR prostate brachytherapy [7, 8] and the expanded use of prostate/pelvic MRI in clinical workups, intraoperative planned HDR brachytherapy utilizing TRUS registered, MRI tumor delineation would provide a reliable and efficient strategy to implant focal prostate brachytherapy at most radiotherapy centers.

## 2. Methods

### 2.1. Selection of Studies

Two separate searches were performed with two slightly different search criteria. A PubMed database search for the terms “focal + brachytherapy + prostate” and for “partial + brachytherapy + prostate” with no limits placed on publication date was completed (10/16/2015). All non-English language manuscripts were then removed at which point the abstracts of the remaining manuscripts were read. Those manuscripts that did not concentrate on focal or focused prostate brachytherapy or were review papers were removed from this pool. Focused treatments where deemed as those in which the whole gland was treated with brachytherapy; however there was a focal boost in the plan in an attempt to deliver more dose to the gross tumor volume (GTV) and/or decrease toxicity to nearby organs at risk (OARs). Inclusion criteria for manuscripts in the final analysis included dosimetric studies that demonstrated treatment and organ at risk dosimetric data of whole gland (HG) compared to focal or focused treatments. Clinical study inclusion criteria included works involving focal or focused brachytherapy that reported treatment toxicity.

### 2.2. Data Extraction and Clinical Endpoints

Data extraction was conducted according to the Preferred Reporting Items for Systematic Reviews and Meta-Analyses (PRISMA) statement [9]. For each study, the following information was extracted: name of the first author, year of publication, number of participants included in analysis, brachytherapy type (HDR versus LDR), RT protocol (whether focal or focused and whether monotherapy or with EBRT), planning imaging modality, and treatment imaging modality. The primary measure of interest for the analysis of dosimetric studies was if OAR and GTV doses were reported for both partial and whole gland treatments which would provide evidence as to whether partial gland therapy is amenable to future definitive salvage therapy (what will be referred to as salvageability). The primary measure of interest for the analysis of clinical studies was toxicity, with secondary interests, if available, in treatment efficacy in regard to survival parameters and treatment dosimetry details. Preliminary analysis indicated that the data provided was insufficient for meta-analysis, thus this work was followed as a PRISMA style systematic review.

## 3. Results

### 3.1. Selection of Manuscripts

**Figure 1:**
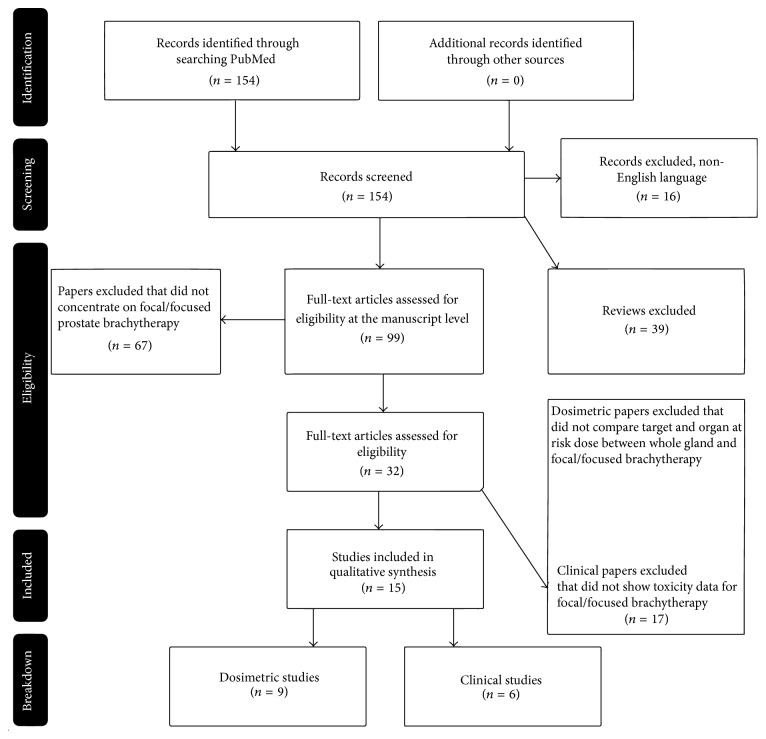
Literature selection process (PRISMA flow diagram).

As outlined in Figure 1, the initial search yielded combined 154 results. Removal of duplicates and non-English language manuscripts reduced this number to 138. All review articles were removed which reduced the pool of papers to 99. The abstracts of these manuscripts were then read to determine if the work concentrated on focal or focused prostate brachytherapy. Resulting 32 papers met these eligibility criteria and were read in entirety for final inclusion. Criteria for dosimetric papers considered for final inclusion were those in which disease target prescription and dose to OARs were characterized between whole gland and focal/focused treatments. Clinical papers were required to demonstrate side effects of focal/focused brachytherapy treatments. In total, 9 dosimetric manuscripts (Table 1) and 6 clinical manuscripts (Table 2) met all eligibility requirements.

### 3.2. Patterns between Selected Dosimetric Manuscripts

The nine dosimetric studies included in this systematic review were published between 2009 and 2015 with a heavy weighting towards more recent publications. There were three different groups/collaborators that contributed more than one publication but looked at different aspects of focal and focused brachytherapy. These included significant work by Mason et al. and Al-Qaisieh et al. [10–13], Kamrava et al. and Banerjee et al. [14, 15], and Peters et al. and Moman et al. [16, 17]. Sample size ranged from 2 to 15 with an average of 9 subjects per study. Of the manuscripts, 3 involved focused treatments [11, 13, 18], one hemigland treatment [14], two focal treatments [17, 19], and three hemi/focal treatments [10, 12, 13], all of which were compared to whole gland therapy (Table 1). One work involved focal treatment as salvage therapy [17], while the rest were initial cancer treatment studies. In regard to planning imaging two manuscripts utilized CT to treat the whole prostate and an arbitrary focus [14, 15]. The remaining works used various MRI sequences alone [11–13, 19], MRI with MR spectroscopy (MRS) [18], or MRI combined with positional information from prostate biopsy [10, 17]. HDR was employed in 6 of the 9 studies [11–15, 17] with LDR seeds in the rest [10, 18, 19]. Two of the HDR studies were plans combined with EBRT [11, 13], while the rest were brachytherapy monotherapy. In regard to HDR monotherapy fractionation, two studies employed a single fraction [12] (one of which was a salvage treatment [17]), while the rest used 6 fractions [14, 15].

Comparison of the target/OAR doses of the partial gland plans to whole gland therapy demonstrated three patterns. Two works using partial gland therapy demonstrated a similar dose to tumor volume and less dose to OARs [14, 19]. Higher dose to the GTV was achieved by partial therapy while maintaining the same dose to the OARs as whole gland in two publications [11, 13]. The remaining 5 manuscripts were able to achieve superior tumor dose with less involvement of the OARs with partial therapy [10, 12, 15, 17, 18]. As previously stated one major theoretical advantage of focal brachytherapy would be to permit definitive RT salvage in the future; therefore manuscripts were studied to determine salvageability. The authors in two of the publications demonstrated that hemi- and focal treatment plans [14, 15] would be amenable to future salvage. Based on typical OAR constraints three other studies [10, 12, 19] could permit further definitive RT given the significant reduction in OAR dosing achieved from partial therapy.

### 3.3. Patterns between Selected Clinical Manuscripts

The six manuscripts that included clinical toxicity data of focal/focused brachytherapy were published between 2002 and 2015 with all but one work completed on or after 2013. The number of patients assessed in the manuscripts ranged from 1 to 20 with a mean number of 7 subjects. Two of the six works utilized a focused [20, 21] rather than focal strategy [16, 22–24] (Table 2). The works were evenly divided between salvage treatments [16, 22, 24] and initial treatments [20, 21, 23] with two focused manuscripts being salvage treatments. Planning was based on MRI imaging [16], MRI combined with biopsy data [20, 24], prostate biopsy mapping [23], and MRI combined with both MRS and biopsy data [22]. Treatment imaging utilized MRI registered TRUS [16, 22], MRI [20], and biopsy mapping with TRUS [21]. One manuscript utilized intraoperative planning and treatment guidance with tissue-type imaging (TTI) based TRUS that enables delineation of tumor volumes from US spectra [23]. Wallace et al. did not specify the method of treatment imaging [24]. All of the works employed LDR brachytherapy.

Dosimetric data was compared to consensus prostate brachytherapy constraints [25] to determine if the focal/focused plan was able to increase tumor dose and decrease dose to OARs. All but one clinical work [23] included in this review published dosimetric data. Both focused works were able to achieve OAR doses typical of a standard whole gland treatment, with increased dose to the GTV [20, 21]. Ennis et al. provided examples of what the dosimetry would have been had they employed whole gland rather than focused plans [21]. The focused plans did slightly increase GTV dose than what would have been accomplished in the whole gland plan. Similarly, Peters et al. with focal brachytherapy were able to achieve higher than standard whole gland prostate GTV dosing while remaining within expected OAR tolerances [16]. The two remaining focal manuscripts were able to achieve significantly lower doses to the OARs, while delivering a dose to the target tumor that is comparable to [24] or greater [22] than what would be achieved with whole gland therapy.

Follow-up ranged from 1 to 60 months with a mean of 23 months. Follow-up duration was not published by DiBiase et al., nor was any disease progression information included [20]. There were no recurrences/failures in three manuscripts with median follow-up of 1 month [24], 12 months [23], and 31.5 months [21]. Hsu et al. had a 5-year biological failure rate as determined by Phoenix criteria of 28.6% [22]. Similarly, Peters et al. demonstrated failure via Phoenix criteria of 15% at 36 months, although half of those were patients that did not respond to therapy initially in regard to PSA [16]. When examining the selected publications' toxicity, the overall reported side effects are no more than would be expected for whole gland brachytherapy [26]. Closer examination of focal plans that significantly reduced OARs demonstrated patterns of decreased toxicity. In particular Hsu et al. with an average V100_rectum_ of 5.5% showed only grade I GI toxicity in 13% of the patients [22], while the single subject in the report of Wallace et al. had no long-term side effects at 3 months in the background of significantly reduced rectal and urethra dose [24]. ED was not significantly changed from pre-RT levels in most studies [16, 20, 23, 24] although two had an increase in the proportion of ED being grade III versus grade I/II from the period before to that after treatment [21, 22], with one work achieving otherwise low side effects and low OAR dose [22].

## 4. Discussion

**Figure 2:**
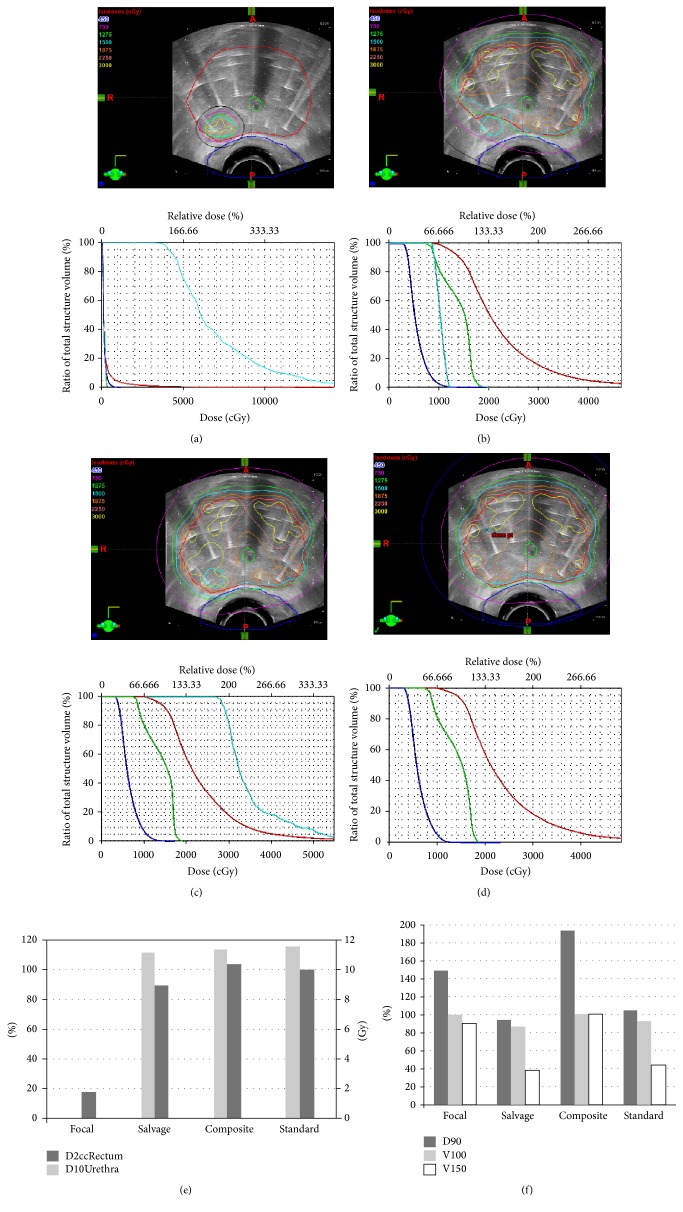
Organ/isodose contours, DVH for focal plan (a), salvage plan (b), composite of focal plan and salvage plan (c), and standard whole gland plan (d) of ideal focal HDR patient. Isodose lines are as follows: Black 450 cGy, Magenta 750 cGy, Green 1275 cGy, Cyan 1500 cGy, Orange 1875 cGy, Dark Pink 2250 cGy, and Yellow 3000 cGy. Target (e) and organ at risk doses (f).

The dosimetric portion of the studies selected in this systematic review demonstrate that a focal plan is attainable in regard to both GTV dosing and decreasing dose to OARs using either LDR or HDR approaches. Furthermore, there is evidence that such partial gland treatments may permit irradiation of prostate tissue outside the GTV for future definitive, salvage RT [14, 15]. Although the number of clinical studies and the number of treated patients are low, focal and focused plans were able to deliver lower than usual dose to OARs with excellent outcomes from a toxicity standpoint. For example, Ennis et al. were able to achieve a significant reduction to the rectal dose that resulted in less GI toxicity than would have been anticipated by a whole gland treatment [22]. Similar to the dosimetric works, the two focal clinical studies with decreased OAR dose would likely be amenable to future salvage RT or surgery [22, 24]. In our own patient population we have found that select low risk patients are appropriate for focal HDR treatments. Figures 2(a), 2(b), and 2(e) demonstrated an example patient where simulation of focal therapy was able to achieve target dose with minimal exposure to OARs and afford future salvage therapy within OAR and target dose constraints. The ultimate composite between the focal and salvage plan is comparable to standard whole gland therapy (Figures 2(c), 2(d), and 2(e) and Table 3). Based on these results and our own experience, the authors of this work feel that sufficient evidence exists to pursue larger clinical studies of focal therapy for well-selected low risk prostate cancer patients.

In the PSA era there is significant lead-time with the detection of early stage disease [6]. For those patients who have extended life expectancy and low risk disease current options are limited to active surveillance, prostatectomy, or definitive RT [5]. The side effects of both whole gland RT and surgery, including the 50% reported incidence of ED [27], dissuade many patients from therapy who may otherwise be active and healthy. On the other hand there are many patients for whom active surveillance provokes too much anxiety, not to mention the morbidity that potential annual biopsy can induce. Essentially, these patients desire a treatment to halt disease progression with low toxicity. The evidence presented in this manuscript suggests that focal brachytherapy can be optimized in low risk patients to deliver a fully therapeutic dose to the GTV while avoiding significant side effects. Further this treatment may possibly be salvageable with either surgery or RT with future disease recurrence. While current definitive treatment with surgery or whole gland RT can be technically salvaged, there are issues with both. After whole prostate RT salvage prostatectomy is difficult to perform on what is an extremely fibrotic post-RT prostate and surrounding tissues [28]. Alternatively, salvage RT is more often treating a significantly greater portion of the pelvis with greater side effects than initial whole gland therapy. Focal brachytherapy may be a more salvageable option that could act as a temporizing bridge to definitive therapy. In doing so, focal brachytherapy could be used to extend a period that is free of morbidity before definitive surgery/RT or in the best-case scenario negate any further treatment.

While none of the clinical papers employed HDR brachytherapy, the technique offers theoretical advantages over LDR for focal treatments [7]. First, the treatment volume can be optimized through inverse planning, which allows compensation for suboptimal needle placement and an overall more reliable dose distribution pattern in regard to the PTV [29]. There are fewer radiation concerns in regard to exposure to physician, nurses, and physicists as the source is delivered remotely. Also, there is no need for postimplant dosimetry, which adds to an already efficient planning/treatment delivery system. With the goal to create a salvageable focal plan to spare OAR and maximize dose to the GTV, the flexibility offered by HDR is more optimal. One significant drawback to HDR as monotherapy is that it is traditionally fractionated, which requires multiple needle insertions, or keeping needles placed for prolonged period of times. However, there is some experience with single fraction HDR [30], which likely would suffice for focal therapy given the dosimetric work of Kamrava et al. and Moman et al. [14, 17].

**Figure 3:**
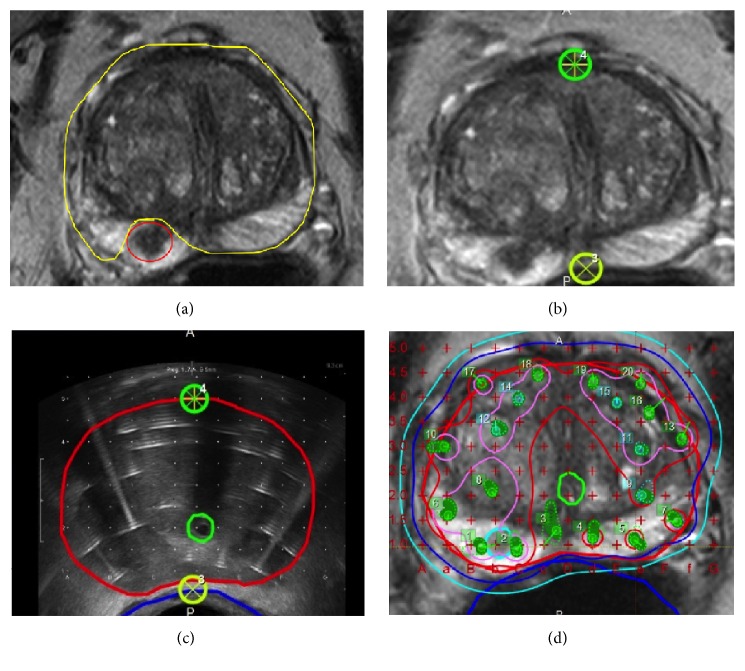
(a) Preprocedural MRI demonstrating GTV (yellow) and nondiseased prostate (red). Preprocedural MRI using two anchor points (green and yellow circles) (b) are able to be registered to intraprocedural TRUS with 2.5 mm grid spacing (c). (d) The resulting merge of the TRUS grid and preprocedural MRI with GTV (thick light blue), urethra (thick solid light green), rectum (thick blue line), and isodose lines (thin lines) for typical whole gland plan using 18 HDR catheters.

An additional benefit of HDR over LDR is its efficient integration with TRUS guidance, allowing a scan-plan-treat environment [8]. TRUS provides improved visualization of the base and apex of the prostate compared to CT and eliminates the need to move the patient from an imaging room to a brachytherapy suite, saving considerable procedure time. However, one significant drawback is that tumors are not readily visualized and as an imaging modality it takes experience to read. There is (TTI) TRUS which has shown success in delineating tumor from normal prostate [21]; however, the imaging requires some skill with interpretation and as a technique is not in wide use. Alternatively, tumors are readily visualized by MRI. However, the long acquisition time and usually off-site location of MRI scanners preclude them from streamlined HDR simulation, planning, and delivery except at a few specialized centers [20]. As shown by many of the manuscripts selected in this review prior pelvic MRI can be registered to the treatment TRUS, which when combined provides efficient intraprocedural tumor delineation. In regard to focal therapy, Mason et al. confirmed that MRI with T2-weighted, diffusion-weighted, and dynamic-contrast-enhanced sequences can accurately detect tumors, negating biopsy mapping [11]. To combine all of the above technologies together, treatment planning systems such as Vitesse Version 3.0 (Varian, Inc. Palo Alto, CA) that supports HDR intraprocedural TRUS guided needle placement, planning, and delivery already exist which as a software can be readily registered with prior MRI data. This registration has been readily performed experimentally at our own institution as shown in Figure 3. Given the increased utilization of pelvic MRI in prostate cancer workup, MRI registered TRUS is an efficient and reliable focal brachytherapy platform that can be adapted to most brachytherapy centers.

There are areas that, based on this systematic review, require further research to validate focal HDR prostate brachytherapy as a reliable technique. Foremost, the current clinical trials are few in number, have short follow-up duration, and are small in sample size. Therefore, it is a plausible that the clinical results presented in this systematic review may not be accurate representations of the toxicity and efficacy from focal plans. Further, the disease stage varied amongst the papers including those with more advanced disease than low risk prostate cancer that would be more applicable to focal treatments. To the knowledge of the authors, there are also no works that directly compare whole gland to focal treatments in regard to patient toxicity and treatment efficacy. These shortcomings would be clarified with a trial of whole gland and focal brachytherapy that is large enough to permit a comparison of toxicity and efficacy, that is, progression free survival, with significant power. In regard to HDR as monotherapy, single fraction therapy would be more attractive to patients than multiple fractions with either prolonged needle placement or repeat needle application. However, there has been very limited publication experience with single fraction HDR monotherapy, thus requiring more research before application as a part of focal brachytherapy strategy.

## 5. Conclusion

In this systematic review we demonstrated dosimetrically that focal brachytherapy could be appropriately applied to prostate tumors while decreasing dose to OARs. Clinical studies were also presented that provided evidence that such treatments decreased toxicity over current whole gland therapy. The decreased toxicity and decreased dose to OARs permit potential salvage RT or salvage prostatectomy if there is future recurrence. Given the indolent nature of prostate cancer when caught at an early stage, focal therapy may serve as a temporary and possible permanent measure for low risk prostate cancer that delays/avoids the significant toxicity such as ED that current definitive therapy has. To this end, HDR brachytherapy allows the dosimetric flexibility required for focal plans. Further, pelvic MRIs, which are increasingly obtained in prostate cancer workup, can be registered to intraprocedural TRUS for an efficient and widely applicable focal brachytherapy platform.

## Figures and Tables

**Table 1:** Summary of dosimetric manuscripts.

Author/year	Focal/focused	Tumor imaging	Treatment imaging	Patient number/technique	Dosing data summary	Sal
Todor et al. 2011 [18]	Focused	MRI/MRS	MRI reg US	*n* = 2 LDR standard versus boost mix of I^125^, Pd^103^, and Cs^131^ seeds	Mixed seeds allowed 20%–66% increase in dose to tumor PTV while decreasing urethra dose by 10% compared to standard single type seed plans	No

Kamrava et al. 2013 [14]	Focal	CT	CT	*n* = 10 HDR 7.25 Gy × 6, WG versus HG plans	WG: D90 112%, V100 97.6%, V150 33.8%, D2cc_Rectum_ 64.1%, D2cc_Bladder_ 67.5%, D2cc_Urethra_ 95.2% HG: D90 108%, V100 98.8%, V150 26.5%, D2cc_Rectum_ 53.1%, D2cc_Bladder_ 55.9%, D2cc_Urethra_ 69.3%	Yes

Mason et al. 2014 [11]	Focused	MRI	MRI reg US	*n* = 15 HDR 15 Gy × 1 fx and then 37.5 Gy in 15 fx EBRT fG versus WG	WG: median D90 17.6 Gy, V150 27.3%, D10cc_Urethra_ 17.2 Gy, D2cc_Rectum_ 8 Gy fG: median D90 20.9 Gy, V150 75.9%, D10cc_Urethra_ 17.4 Gy, D2cc_Rectum_ 9 Gy	No

Mason et al. 2014 [12]	Focal	MRI	MRI reg US	*n* = 9 HDR 19 Gy × 1, WG versus HG versus FG	WG: D90 20.4 Gy, V100 97.9%, D2cc_Rectal_ 20.3 Gy, D10_Urethra_ 12.5 Gy HG: D90 22.2 Gy, V100 98.1%, D2cc_Rectal_ 19.7 Gy, D10_Urethra_ 9.8 Gy FG: D90 23.0 Gy, V100 98.2%, D2cc_Rectal_ 9.2 Gy, D10_Urethra_ 4.6 Gy	Yes

Polders et al. 2015 [19]	Focal	MRI	MRI reg US	*n* = 15 LDR I^125^ 145 Gy × 1 fx, WG versus FG	WG: D90_GTV_ 198 ± 44 Gy, V100_GTV_ 94% (89–100), D2cc_Rectal_ 99 ± 19 Gy, D10_Urethra_ 214 ± 21 Gy, FG: D90_GTV_ 195 ± 60 Gy, V100_GTV_ 94% (71–100), D2cc_Rectal_ 37 ± 21 Gy, D10_Urethra_ 79 ± 33 Gy,	Yes

Banerjee et al. 2015 [15]	Focal	CT	CT	*n* = 5 HDR 7.25 Gy × 6 fx, WG versus HG versus 1/6 gland	WG: D90 109.3% V100 98.7 D2cc_Bladder_ 64.8%, D2cc_Rectal_ 65.3%, D1cc_Urethra_ 103.8% V75_Urethra_ 75% HG: D90 112.7% V100 97.8 D2cc_Bladder_ 56.2% D2cc_Rectal_ 54.2% D1cc_Urethra_ 86.5% V75_Urethra_ 57.1% 1/6 gland: D90 114.7% V100 97.4 D2cc_Bladder_ 24.7%, D2cc_Rectal_ 32.8%, D1cc_Urethra_ 52.1% V75_Urethra_ 14.5%	Yes

Al-Qaisieh et al. 2015 [10]	Focal	MRI/biopsy mapping	MRI reg US	*n* = 14 LDR I^125^ 145 Gy × 1 fx WG versus HG versus FG	WG: D90 181.3 Gy, V100 99.8%, D2cc_Rectal_ 107.5 Gy, D10_Urethra_ 205.9 Gy HG: D90 195.7 Gy, V100 97.8%, D2cc_Rectal_ 77.0 Gy, D10_Urethra_ 191.4 Gy FG: D90 205.9 Gy, V100 99.8%, D2cc_Rectal_ 42.7 Gy, D10_Urethra_ 92.4 Gy	Yes

Mason et al. 2015 [13]	Focused	MRI	MRI reg US	*n* = 15 HDR 15 Gy × 1 fx followed by EBRT 37.5 Gy in 15 fx. WG versus fG	Median values for PTV_Focused_ in WG: D90 18.3 Gy, V150 33.7 Gy, V200 8.9 Gy, D10_Urethra_ 17.1 Gy, D2cc_Rectum_ 8.4 Gy Median values for PTV_Focused_ in fG: D90 24.3 Gy, V150 77.2 Gy, V200 30.2 Gy, D10_Urethra_ 17.2 Gy, D2cc_Rectum_ 8.9 Gy	No

Moman et al. 2010 [17]	Focal	MRI/biopsy mapping	NR	*n* = 3 HDR 15 Gy × 1 fx WG versus FG post-RT salvage plans	WG: V100_GTV_ 25–53%, D2cc_Rectal_ 5.0–7.2 Gy, D2cc_Bladder_ 6.5–12 Gy FG: V100_GTV_ 93–100%, D2cc_Rectal_ 2.5–6.4 Gy, D2cc_Bladder_ 1.4–4.3 Gy	NA

MRS: magnetic resonance spectroscopy, reg: registered, Sal: salvageable, WG: whole gland, HG: hemigland, FG: focal gland plan, fG: focused gland plan, NR: not reported, and NA: not applicable.

**Table 2:** Summary of clinical manuscripts.

Author/ year	Focal/ focused	Tumor/ treatment imaging	Technique (HDR/LDR)	Dosimetry	Symptoms/outcome	Salv.
DiBiase et al. 2002 [20]	Focused	MRI, Biopsy mapping/MRI	*n* = 14 LDR I^125^ 145 Gy × 1 fx, LR disease, 130% boost to gross tumor	V100 95% (89–98%) Dmax_Urethra_ 160% (range 131–220%) Dmax_Rectal_ 110% (range 74–150%)	FU duration not recorded, progression not reported GU toxicity grade I 87%, grade II 53% No urinary incontinence, no rectal toxicity	No

Hsu et al. 2013 [22]	Focal	MRI, MRS, biopsy mapping/MRI reg US	*n* = 15 LDR I^125^ 144 Gy × 1 fx. Post LDR focal salvage, TRUS confirmed disease, 1-2 foci.	D90_CTV_ 187.5 Gy (107.5–247.5) V100_CTV_ 99.0% (91.7–100.0) V100_Rectum_ 5.5% (0.1–18.7)	5 y FU, recurrence (Phoenix criteria): 1 y 0%, 2 y 0%, and 3 y 62.7% Pre-GI grade I, 13.3%, grade II, 6.7%, post-GI grade I, 13.3, grade II, 0% Pre-GU grade I, 33.3%, grade II, 60.0%, grade III, 6.7% Post-GI: grade I, 6.7%, grade II, 60.0%, grade III, 33.3% Pre ED: grade I, 26.7%, grade II, 53.3%, grade III, 0% Post ED: grade I, 13%, grade II, 66.7%, grade III, 13.3%	Yes^*∗*^

Barret et al. 2013 [23]	Focal	biopsy mapping/TRUS	*n* = 12 LDR I^125^ compared to other focal techniques in LR disease	NR	FU to one year, PSA change median 6.2 (5–7.9) baseline to 2.5 (0.9–4.4) at 1 year Median IPSS_baseline_ 3 (1–7), median IPSS_1 yr_ 7 (2–12) Median IIEF-5_baseline_ 21 (10–25), median IIEF-5_1 yr_ 14 (8–24)	NA

Wallace et al. 2013 [24]	Focal	MRI, biopsy mapping/NR	*n* = 1 LDR Pd^103^ 124 Gy × 1 fx Single patient s/p EBRT 75 Gy for IR disease, Salvage at 4 years	D90_GTV_ 100%, V100_GTV_ 100% V100_rectal_ 0.00cc D30_urethra_ 54.52%	Median FU 1 month, PSA at one year 0.52 and decreasing Postsalvage: no ED, nocturia 0-1x, 1-2 hr daily voiding AUA 21 from baseline of 5, SHIM score 22 (No ED) 3 months: GU and GI symptoms back to baseline	Yes^*∗*^

Peters et al. 2014 [16]	Focal	MRI/MRI reg US	*n* = 20 LDR, LR, IR & HR, I^125^ 144 Gy × 1 fx focal salvage, 35% post LDR, 65% post EBRT, TRUS confirmed unilateral disease 2 y post RT	D90_GTV_ 198 Gy (150–330) D2cc_rectum_ 68 Gy (18–96) D0.1cc_rectum_ 133 Gy (69–207) D10_urethral_ was 132 Gy (100–240)	Median FU 36 months, recurrence (Phoenix criteria): 15% (3 of 6 no initial response) Presalvage ED: grade II, 50%, grade III, 25%, 3 yr ED: grade II, 35%, grade III, 20% Presalvage GI grade I, 45%, grade II, 10%, 3 yr GI grade I, 13.3%, grade II, 15% Presalvage GU grade I, 25%, grade II, 10%, 3 yr GU grade I, 5%, grade II, 15% Only one patient: grade III toxicity (GU) at 3 years FU	No^**^

Ennis et al. 2015 [21]	Focused	TTI TRUS/TRUS	*n* = 14 LDR Pd-103, prospective IR patients with Gleason ≤ 6. Total prostate treated with dose painting (GTV goal D100 200% and same OAR constraints as typical plan)	Standard whole gland plan: V200_GTV_ 49% V150_Urethra_ 1% D1CC_Rectum_ 51% Focused plan: V200_GTV_ 97% V150_Urethra_ 11% D1CC_Rectum_ 60%	Biological recurrence: 0% at 31.5 months Acute (less than 6 months), late (greater than 6 months) Acute proctitis grade I, 23%, grade II, 0%, late proctitis grade I, 0%, grade II, 17% Acute urgency/frequency grade I, 54%, grade II, 46% Late urgency/frequency grade I, 58%, grade II, 17% Acute ED: grade I, 31%, grade II, 54%, grade III, 0% Late ED: grade I 33%, grade II, 42%, grade III, 17% No grade III toxicity	No

FU: follow-up, GU: genitourinary, GI: gastrointestinal, Salv.: salvageable, LR: low risk, IR: intermediate risk, HR: high risk, reg: registered, ED: erectile dysfunction, NR: not reported, TTI: tissue-type imaging, IPSS: International Prostate Symptom Score, IIEF-5: 5-item version of the International Index of Erectile Function.

^*∗*^Would be salvageable given data if not salvage therapy.

^*∗∗*^Would not be salvageable given data if not salvage therapy.

**Table 3:** Experimental dose to target and OAR between focal, salvage, and whole gland HDR brachytherapy.

Parameter	Focal	Salvage	Composite	Standard
D2cc_Rectum_ (Gy)	1.76	8.90	10.29	9.98
D10_Urethra_ (%)	0.00	111.66	113.85	115.64
D90_PTV_ (%)	149.30	94.15	193.9	104.54
V100_PTV_ (%)	100.00	86.51	100.00	92.70
V150_PTV_ (%)	89.70	37.89	100.00	43.72
